# Conservation genomics of the wild pumpkin *Cucurbita radicans* in Central Mexico: The influence of a changing environment on the genetic diversity and differentiation of a rare species

**DOI:** 10.1007/s10265-024-01552-1

**Published:** 2024-07-08

**Authors:** Jaime Gasca-Pineda, Brenda Monterrubio, Guillermo Sánchez-de la Vega, Erika Aguirre-Planter, Rafael Lira-Saade, Luis E. Eguiarte

**Affiliations:** 1 Departamento de Ecología Evolutiva, Instituto de Ecología, Universidad Nacional Autónoma de México, Circuito Exterior s/n Anexo al Jardín Botánico, Ciudad de México, 04510 México; 2 Unidad de Biotecnología y Prototipos, Facultad de Estudios Superiores Iztacala, Universidad Nacional Autónoma de México, Av. De Los Barrios 1, Col. Los Reyes Iztacala, Tlalnepantla, Estado de México 54090 México

**Keywords:** Crop wild relative, Genetic structure, Outlier test, Genotype by sequencing, Population expansion

## Abstract

The genetic diversity found in natural populations is the result of the evolutionary forces in response to historical and contemporary factors. The environmental characteristics and geological history of Mexico promoted the evolution and diversification of plant species, including wild relatives of crops such as the wild pumpkins (*Cucurbita*). Wild pumpkin species are found in a variety of habitats, evidencing their capability to adapt to different environments. Despite the potential value of wild *Cucurbita* as a genetic reservoir for crops, there is a lack of studies on their genetic diversity. *Cucurbita radicans* is an endangered species threatened by habitat destruction leading to low densities in small and isolated populations. Here, we analyze Genotype by Sequencing genomic data of the wild pumpkin *C. radicans* to evaluate the influence of factors like isolation, demographic history, and the environment shaping the amount and distribution of its genetic variation. We analyzed 91 individuals from 14 localities along its reported distribution. We obtained 5,107 SNPs and found medium-high levels of genetic diversity and genetic structure distributed in four main geographic areas with different environmental conditions. Moreover, we found signals of demographic growth related to historical climatic shifts. Outlier loci analysis showed significant association with the environment, principally with precipitation variables. Also, the outlier loci displayed differential changes in their frequencies in response to future global climate change scenarios. Using the results of genetic structure, outlier loci and multivariate analyses of the environmental conditions, we propose priority localities for conservation that encompass most of the genetic diversity of *C. radicans*.

**Supplementary Information:**

The online version contains supplementary material available at 10.1007/s10265-024-01552-1.

## Introduction

The amount and distribution of genetic variation in natural populations is determined by evolutionary mechanisms such as selection, genetic drift, and gene flow, along with their interaction with factors like population isolation, historical demographics, and anthropogenic influence (Allendorf et al. 2012; Chung et al. 2023). Consequently, the current patterns of genetic diversity are the result of a mixture of recent and historical factors. For example, the resulting genetic diversity driven by genetic drift may be influenced by the interaction of population contractions with genetic isolation, and the intensity of gene flow (Slatkin 1981). However, genetic variation can also be altered by local adaptation, leading to divergence in specific genes as a response to changing environments (Davis and Shaw 2001; Hoffmann et al. 2021; Leimu and Fischer 2008). Hence, disentangling the patterns of genetic diversity contributes to our understanding of the evolution and ecology of species, and the generated information may be valuable for conservation (Hoffmann et al. 2021; Nielsen et al. 2023; Theissinger et al. 2023).

Mexico has been recognized as a megadiverse country harboring about 10% of the worldwide biodiversity (Dirzo and Saruhkán 1992). Among the explanations of this diversity are the geological history, topographic heterogeneity, diversity of climates, and the geographical location, resulting in a complex mosaic of environmental conditions (Piñero et al. 2008; Dirzo and Saruhkán 1992). This environmental heterogeneity lead to the origin and diversification of several plant species, including wild relatives of economically valuable plants, such as maize, beans, agave, and pumpkins (Aguirre-Liguori et al. 2019; Eguiarte et al. 2021; Kraft et al. 2014; Lira 1996; Moreno-Letelier et al. 2020). Among these species, the *Cucurbita* genus comprises around 15 species, most of them endemic to Mexico and Mesoamerica (Goettsch et al. 2021; Lira and Caballero 2002). *Cucurbita* plants inhabit diverse environments, and have been traditionally divided according to their ecology and habitat in xerophytic and mesophytic species (Lira et al. 2016). Also, the genera include all the commercial pumpkins and squashes (*C. pepo* L., *C. moschata* Duchesne, *C. maxima* Duchesne, *C. argyrosperma* Huber, *C. ficifolia* Bouché). The principal pollinators of cucurbit flowers are bees from the genus *Apis*, *Eucera and Bombs* (Pfister et al. 2017), and introgression among wild and domesticated pumpkins has been reported (Barrera-Redondo et al. 2021; Lira et al. 2016). Even though bees are less attracted to the pollen from wild *Cucurbita* due to protein concentration differences (Vaudo et al. 2020), it has been proposed that conserving wild cucurbit plant populations is essential to secure pollination services in domesticated cucurbits (Glasser et al. 2023).

Despite the relevance of studying the genetic diversity of crop wild relatives for the discovery of useful genetic variants for crop improvement (Aguirre-Liguori et al. 2019; Barrera-Redondo et al. 2021; Goettsch et al. 2021; Kates 2019), most studies that includes wild pumpkins have focused on gene flow between crops and their wild relatives (Aguirre-Dugua et al. 2023; Barrera-Redondo et al. 2021; Kates et al. 2021; Martínez-González et al. 2021; Papa and Gepts 2004; Sánchez-de la Vega et al. 2018; Wilson et al. 1994), resulting in a lack of population-level studies for wild *Cucurbita* taxa. Among the wild squashes, *Cucurbita radicans* Naudin is a xerophytic species endemic to the Trans-Mexican Volcanic Belt. *C. radicans* inhabits principally grasslands and disturbed areas associated with crops like maize and other *Cucurbita* species (Lira 2001; Lira et al. 2009). Its geographic distribution includes an elevation range from 950 to 3000 m.a.s.l. with an average annual temperature from 12.5 °C to 28.4 °C (data from WorldClim: www.worldclim.org; Fick and Hijmans 2017). Despite its relatively large geographical distribution, its current area of occupancy is very narrow, mostly with fragmented and isolated populations (Aragón et al. 2020; Lira and Rodríguez-Arévalo 2006). Due to the current size of its populations and habitat reduction, *C. radicans* is listed under the EN (Endangered) category of the Red List of the IUCN (Aragón et al. 2020; www.iucnredlist.org). Moreover, the records of this species and herbarium specimens are scarce (Aragón et al. 2020; Lira 1996). Apart from studies about its basic biology and distribution, there is no detailed information about the genetic diversity and structure of its populations (Aragón et al. 2020; Lira 2001; Lira and Caballero 2002). On the other hand, *C. radicans* has features like drought tolerance and tuberous roots, making it an interesting model to evaluate adaptations to the environment (Calderón and Rzedowski 2001; Mejía-Morales et al. 2021).

According to Castellanos-Morales et al. (2018), the current distribution and diversity of wild pumpkins in Central Mexico can be attributed to the formation of the Trans-Mexican Volcanic Belt 3 − 7.5 million years ago (mya) (Gómez-Tuena et al. 2007). As a result of this geological event, wild squash populations were isolated, and subsequently adapted to different environmental conditions. Furthermore, the authors suggest that Pleistocene climate changes caused shifts in the distribution ranges of wild squash populations. In particular, the range of suitable habitat for *C. radicans* was very limited during the Last Inter Glacial (LIG, 120,000 years ago), and it wasn’t until the Last Glacial Maximum (∼ 21,000 years ago) that the environment started to become suitable for the species, leading to a rapid expansion of its range. However, following this expansion, the wild pumpkins suffered habitat range fragmentation due to the extinction of their natural dispersers during the Holocene (Kistler et al. 2015). This leaves important questions about the amount of genetic diversity and structure of its populations, how this species may cope with future climate change, and about proposing conservation areas for this endangered species.

In this study, we used Genotyping by Sequencing (GBS) genomic data to evaluate the factors that shaped the amount and distribution of genetic diversity of *C. radicans* in Central Mexico. Considering the species’ environmental range, its fragmented and small populations, and the historical environmental changes in Central Mexico, we have predicted the following results: First, as a consequence of the discontinuous distribution due to the absence of dispersers (Kistler et al. 2015) and the reduced size of local populations due to recent habitat destruction (Aragón et al. 2020), we expect high genetic structure among localities with low to moderate genetic diversity within populations due to the loss of genetic diversity by genetic drift. Second, due to the variety of environmental conditions where *C. radicans* inhabits, we anticipate detecting outlier loci associated with the environment due to local adaptation (Frichot et al. 2013). Third, if genetic diversity is associated with the environment, we expect allele frequency changes in outlier loci in response to a future global climate change scenario. Fourth, we anticipate signals of historical demographic growth in *C. radicans* populations, consistent with the historical range expansion documented by Castellanos-Morales et al. (2018). To test these predictions, we collected and genotyped individuals using the available *C. radicans* distribution records along its reported distribution range. We estimated contemporary and historical gene flow, applied outlier loci tests and evaluated the impact of future climate change on allele frequencies. Finally, we performed a historical demographic analysis to assess the impact of the historical climate shifts in Central Mexico on the genetic variation of *C. radicans*. With the results, we proposed zones with high genetic diversity to define priority areas for conservation.

## Methods

### Sample collection, DNA extraction and genotyping

**Fig. 1:**
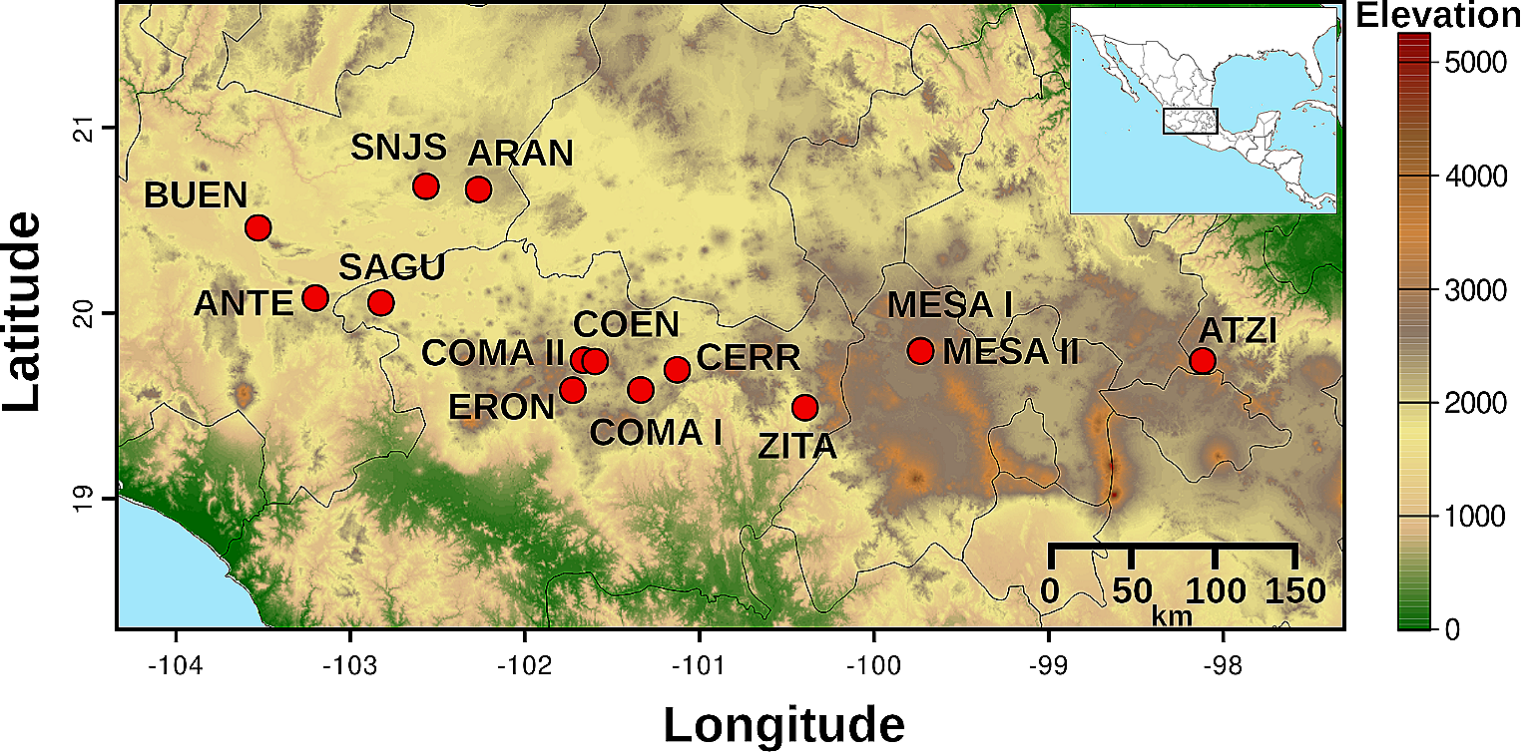
Geographic distribution of the 14 sampling locations from *Cucurbita radicans* in Central Mexico. The color scale represents elevation in meters

Between the years 2016 and 2019, we collected 94 samples of *C. radicans* from 14 localities along their reported distribution with an elevation range of 1,505 to 2,804 m above sea level (Fig. 1, Table S1); further, our sample from San José Atzintlimeya (ATZI) locality represented a new record outside the previously reported distribution for the species (Aragón et al. 2020; Lira and Rodríguez-Arévalo 2006). Fruits were collected from different plants, allowing a minimal distance of 50 m among individuals to minimize the possibility of collecting the same plant. The taxonomic identification of all samples was verified by Cucurbitaceae taxonomic experts (Dr. Guillermo Sánchez-de la Vega in 2018 and 2019 comm. pers; Dr. Rafael Lira-Saade in 2019 comm. pers.).

One seed per plant was germinated in the greenhouse, and total genomic DNA was extracted from fresh leaf tissue from seedlings (except for the sample Atzi1, extracted from the fruit peduncle) using a modified version of the CTAB extraction method (Doyle and Doyle 1987; Klimova et al. 2022). We checked the integrity of the DNA using 1.5% GelRed stained agarose gels and a NanoDrop spectrophotometer (230/260 and 260/280 ratios > 1.8) (Thermo Fisher Scientific, Waltham, MA, United States). The concentration was assessed with a Qubit 3.0 fluorometer dsDNA Broad Range assay (Life Technologies, USA). DNA was genotyped with GBS at the Genomics Center of the University of Minnesota (https://genomics.umn.edu/), using the NspI and BfuCI/Sau3AI restriction enzymes (this set of enzymes has been used successfully in another *Cucurbita* species; see Aguirre-Dugua et al. 2023) with fragment size selection of 200–300 base pairs. Sequencing was performed with an Illumina Novaseq instrument (1 × 100 bp, SE). Raw fastq data is available in the NCBI under the BioProject ID PRJNA976254, and accession numbers SRR24737162-SRR24737255.

### Raw reads filtering and genotyping

To trim the reads by quality and to remove adapters, we used trimmomatic v0.35 (Bolger et al. 2014) with the Nextera adapters included in the program. We used the parameters 2:30:10, a sliding window trimming of 4:20, and keeping a fragment minimal length of 50 bp. Then, read quality was visually inspected using the Bioconductor package ShortRead v1.46.0 (Morgan et al. 2009) in R v4.2.1 (R Core Team 2022). Loci were obtained using ipyrad v0.955 (Eaton and Overcast 2020). As a part of the ipyrad pipeline, we applied a second quality and adapter filtering using cutadapt v3.2 (Martin 2011). We considered a limit of phred scores of 20 at more than five bases per read, and a minimum depth of six was required for statistical base calling. We used the reference ensemble method, using the *Cucurbita maxima* genome (Sun et al. 2017) downloaded from the Cucurbit Genomics Database (CuGenDB, http://cucurbitgenomics.org/; Zheng et al. 2019). We used this genome as reference because it was the closest *Cucurbita* full assembled genome at the time.

The allele post-filtering was carried out using VCFtools v0.1.16 (Danecek et al. 2011) with a thinning of 450 pb to select only one marker per locus, a minimum mean individual depth of 15x, a filter for binary markers, a maximum missing data per loci of 0.10, a Minimum Allele Frequency (MAF) of 0.05, and a Hardy-Weinberg threshold value of 0.05. Finally, to reduce bias due to linkage disequilibrium, we used plink v1.9 (Purcell et al. 2007) to prune loci with high levels of linkage disequilibrium, using a window and step size of 100 base pairs (bp), and *r*^2^ threshold of 0.25. To evaluate the possible bias due to paralogous copies, we performed an additional *denovo* run of ipyrad as suggested by McCartney-Melstad et al. (2019). The objective of this pipeline is to optimize the ipyrad settings that maximize the proper separation of paralogous, minimizing oversplitting of loci. While we found a lower number of loci (3,541 SNPs), we observed similar levels of diversity (*H*_*E*_ = 0.229, *F*_*IS*_ = 0.1421), indicating a negligible influence of paralogous copies in our referenced pipeline.

### Population genetics analysis

We used the mlg function of the R package poppr v2.9.2 (Kamvar et al. 2014) to identify duplicated genotypes due to clonal reproduction or close parentage. Then, we estimated the basic summary statistics *H*_*E*_ and *H*_*O*_, using the adegenet package (Jombart 2008; Jombart and Ahmed 2011). *F*_*IS*_ and its significance was estimated using the R package genepop (Rousset 2008) using the *ad-hoc* tests for excess or deficiency of heterozygotes. We obtained the relatedness statistic proposed by Manichaikul et al. (2010) to assess the level of parentage of individuals using vcftools; this statistic estimates the degree of relatedness among pairs of individuals with a range from zero (non-related) to 0.5 (monozygotic twin). This analysis allows us to explore the degree of dispersion of gametes of this species.

To detect statistically outlier loci (i.e., potentially under selection), we performed ecological association tests based on a latent factor mixed model (LFMM, Frichot et al. 2013), using the LEA v3.12 R package (Frichot and François 2015). We opted for this method because it does not require an *a priori* definition of genetic groups, as *F*_*ST*_-based methods do. The method considers that population structure is shaped by shared demographic history and performs tests for association between allele frequencies and environmental predictors (De Villemereuil et al. 2014; Frichot et al. 2013; Lotterhos and Whitlock 2015). We ran the analysis using the five environmental variables selected (Bio.2, Bio.8, Bio.12, Bio.14 and Bio.15, see below). To choose the significant outliers, we implemented a False Recovery Rate (FDR) analysis using the qvalue R package (Storey et al. 2020), with a threshold value *q* < 0.1. All posterior genetic diversity analyses were implemented excluding the outlier loci.

To infer the putative function of the outlier loci, the significant SNPs were mapped to the reference genome and extracted 500 bp in both directions in order to get a sequence of 1,000 bp. The resulting sequences were aligned against the UniProt database (https://www.uniprot.org/), restricting the search to the Cucurbitaceae family with an *e*-value threshold of 1 × 10^−5^.

### Genetic structure

As a first inspection of genetic structure, we estimated the pairwise Nei *F*_*ST*_ (Nei 1987) among sampling localities using the hierfstat v0.5 package (Goudet and Jombart 2022). Its significance was assessed by bootstrapping over loci 500 times, and if the 95% interval did not reach zero, we considered the value significant. As our initial analyses of the genetic diversity revealed an intricate genetic structure, we implemented three distinct methods to identify the number of genetic groups. To evaluate the patterns of genetic differentiation among localities using spatial information, we used the R package conStruct v1.0.4 (Bradburd et al. 2018). This method uses the allele frequencies genotyped across pairs of samples and computes the decay of allelic covariance among them while considering spatial information. For this analysis, we used the locations as sampling units and implemented the spatial and non-spatial models. We ran each model considering *K* values from 1 to 10 (each value of *K* representing the possible number of genetic groups), for 1 × 10^6^ steps with ten independent chains. To assess for MCMC convergence, we inspected the trace plots for a stable behavior along chains using the R package coda (Plummer et al. 2006). To select the *K* value that better explained the genetic differentiation, we used the cross-validation test (CV), along with the layer contribution metric of overall covariance to the evaluated *K* values. To select which model (spatial vs. non-spatial) explained better the observed genetic structure, we performed a *t*-test between the top cross-validation values, as suggested by the package authors. The final admixture values were obtained using the median value over ten independent chains. We used adegenet to perform a Principal Components Analysis (PCA) to visualize the possible aggregation of individuals based on their similarity or differences in their allelic composition without *a priori* information of the locality of origin. We also used Admixture v1.3.0 (Alexander et al. 2009) to estimate the number of groups (*K*) based on the individual ancestries (*Q*). The optimal value of *K* was determined by the lowest 100-fold CV error with the number of genetic groups *K* ranging from 1 to 20. As we observed discrepancies in the optimal value of CV among runs, we ran the analysis 100 times and recovered the top optimal values of CV. Once the genetic groups were defined, we re-estimated the pairwise *F*_*ST*_ to evaluate the genetic differentiation between groups using the hierfstat package.

### Recent and historical gene flow

To investigate gene flow among *C. radicans* genetic groups, we implemented two complementary methods. First, we used BayesASS v3.0.4 (Mussmann et al. 2019) to estimate contemporary migration rates (over the last 20 generations) among groups, using 5 × 10^7^ MCMC iterations sampling every 5,000 steps. To check for convergence of the MCMC, we plotted the trace files using the R package coda. Second, to estimate the historical migration rates, we used the coalescent-based software migrate-n v5.0.4 (Beerli 1998; Beerli and Palczewski 2010; Beerli et al. 2019). The program estimates the parameters *θ* defined as 4*N*_e_*u*, the effective size multiplied by the mutation rate, and *M*, defined as the immigration rate per generation. We calculated the number of migrants per generation (*Nm*) using the *θ* values with the equation: *Nm* = [(*θ*x * *M*y→x) / 4]), where *My→x* is defined as the genetic flow from population *y* to population *x*. We used starting values of *θ* and *M* from uniform priors (for *θ*, minimum = 0.0, maximum = 0.1, delta = 0.01; for *M*, minimum = 0.0, maximum = 1000.0, and delta = 100.0). Parameter space was searched using five parallel chains with a static heating scheme (temperatures: 1.0, 1.5, 3.0, 1,000,000) with five replicates. We ran each chain for 10 million generations and sampled every 100 generations, and the first 10% steps were discarded as burn in. We inspected histograms of estimated *θ* and *M* posterior values (bin number = 1500) to assess convergence and to check that the distribution of priors was adequate. In general, *Nm* values < 1 suggest that the effect of genetic drift is greater than the effect of gene flow, suggesting genetic isolation of populations.

### Environmental variable selection and prediction of allele frequencies

We generated an environmental data set using the climatic variables from the WorldClim database (Fick and Hijmans 2017). To reduce multicollinearity among variables, we performed a Variance Inflation Factor (VIF) test and retained the set of variables with a VIF value below 10 using the package usdm (Naimi et al. 2014). Then, we performed a PCA and selected the top five contributing variables from the first two principal components (73% overall variance). The final data set included two variables associated with temperature (Bio.2, Mean Diurnal Range, and Bio.8, Mean Temperature of Wettest Quarter), and three with precipitation (Bio.12 Annual Precipitation, Bio.14 Precipitation of Driest Month, and Bio.15 Precipitation Seasonality).

To evaluate the change in outlier loci frequencies under a future global climate change scenario, we used AlleleShift v1.5 R package (Kindt 2021). The AlleleShift method is a Redundancy Analysis that uses the allele frequencies as response data and the current environmental data as explanatory variables to calibrate a baseline model. Then, the model predicts the behavior of allele frequencies using the temporal projected changes of the same set of bioclimatic variables. We used the Coupled Model Intercomparison Project Phase 6 (CMIP6, https://wcrp-cmip.org/cmip-phase-6-cmip6/) downscaled projections considering the extreme (ssp585) Shared Socio-economic Pathways of the Community Climate System Model (CCSM) projected to the year 2100. Plots were generated using the function shift.dot.ggplot provided in the package. (https://github.com/cran/AlleleShift).

### Historical demographic trends

We used the Multiple Sequentially Markovian Coalescent implemented in MSMC2 v2.1 (Schiffels and Durbin 2014; Schiffels and Wang 2020) to infer *C. radicans* demographic history and to approximate the time of separation of the genetic groups. This program models ancestral relationships using the coalescent under recombination along multiple phased genome sequences in isolated populations to fit a demographic model to the data. When individuals come from two different populations (in our case the genetic groups), the model estimates the relative Cross-Coalescence Rate (rCCR) between lineages sampled within each population and between lineages sampled across the two populations. A value of rCCR close to 1 indicates that the two populations were one population at that time, if the rCCR is close to zero, the two populations are considered as two isolated units, and a rCCR value of 0.5 indicates the split time between the two populations. Briefly, we used the bam files generated by the ipyrad pipeline, and generated single-chromosome haplotype input files using custom scripts. We used shapeit v2.r904 (Delaneau et al. 2012) to statistically phase the chromosome haplotypes. As the authors do not recommend using more than 16 haplotypes per run, we performed a random subsampling from the total of individuals, generating 100 random sets from each genetic group to include most of the genetic diversity in our sample. To date the time of demographic change, MSMC2 requires a genomic mutation rate per site per generation, which may be difficult to approximate in non-model organisms. Due to this, we expressed population size as the scaled effective size defined as *λ* − 1 = *N*_*i*_ / *N*_*0*_ where *N* is *θ* / *4µ* at the time interval *i* (*θ* is the Watterson estimator, Watterson 1975). Time is expressed as t / (*µ/g*) where *µ* is the mutation rate per site per generation and *g* the generation time. According to our observations in the field, we considered one year as a reasonable generation time for *C. radicans*. However, to associate the population size changes (i.e. when the effective size was not constant) to a time interval in years, we implemented a mutation rate range from 5 × 10^−8^ to 5 × 10^−9^ mutations per site per generation substituting in the above described equation. This interval encompasses the rate range reported for species like *Panicum hallii* (6.5 × 10^−8^, Lovell et al. 2018) and *Vitis vinifera* (2.5 × 10^−9^, Zhou et al. 2017).

### Conservation priority areas

Finally, we used the resulting genetic variation data to propose the priority localities for *C. radicans* conservation, using the following criteria: (a) To represent most of the genetic diversity, the selected localities must be included in the four quadrants of individual-level genetic diversity PCA, (b) Likewise, the localities have to encompass four quadrants of the environmental PCA, (c) All genetic components from the conStruct and Admixture analysis must be included, and (d) At least a 90% of the SNPs should be polymorphic from the total sample. Using these four criteria, we ensure to preserve the genetic diversity, including the potential adaptive genetic variation.

## Results

We obtained 5,107 biallelic loci for 91 individuals with unique genotypes from 14 localities of *C. radicans* from the 94 sampled fruits. The LEA test identified 47 outlier loci, hence, successive analyses were estimated using only the 5,060 neutral loci. The overall genetic diversity was *H*_*E*_ = 0.268 (SD = 0.126), *H*_O_ = 0.254 (SD = 0.115), and we obtained a low but significant value of *F*_*IS*_ of 0.05 (*p* < 0.0001). Among localities, the genetic diversity ranged from *H*_*E*_ = 0.1604 (MESA II) to *H*_*E*_ = 0.2653 (COMA II). However, in terms of diversity by locality, we observed that genetic diversity was correlated with the sample size, as larger sizes presented higher diversity (*P* = 0.012, linear model). However, it is important to note that our sample sizes were determined by the abundance of the species at each sampling location, in particular in the ATZI, SAGU, ERON, and MESA where we collected from one to three samples. Summary statistics for localities are reported in Table S2.

**Fig. 2:**
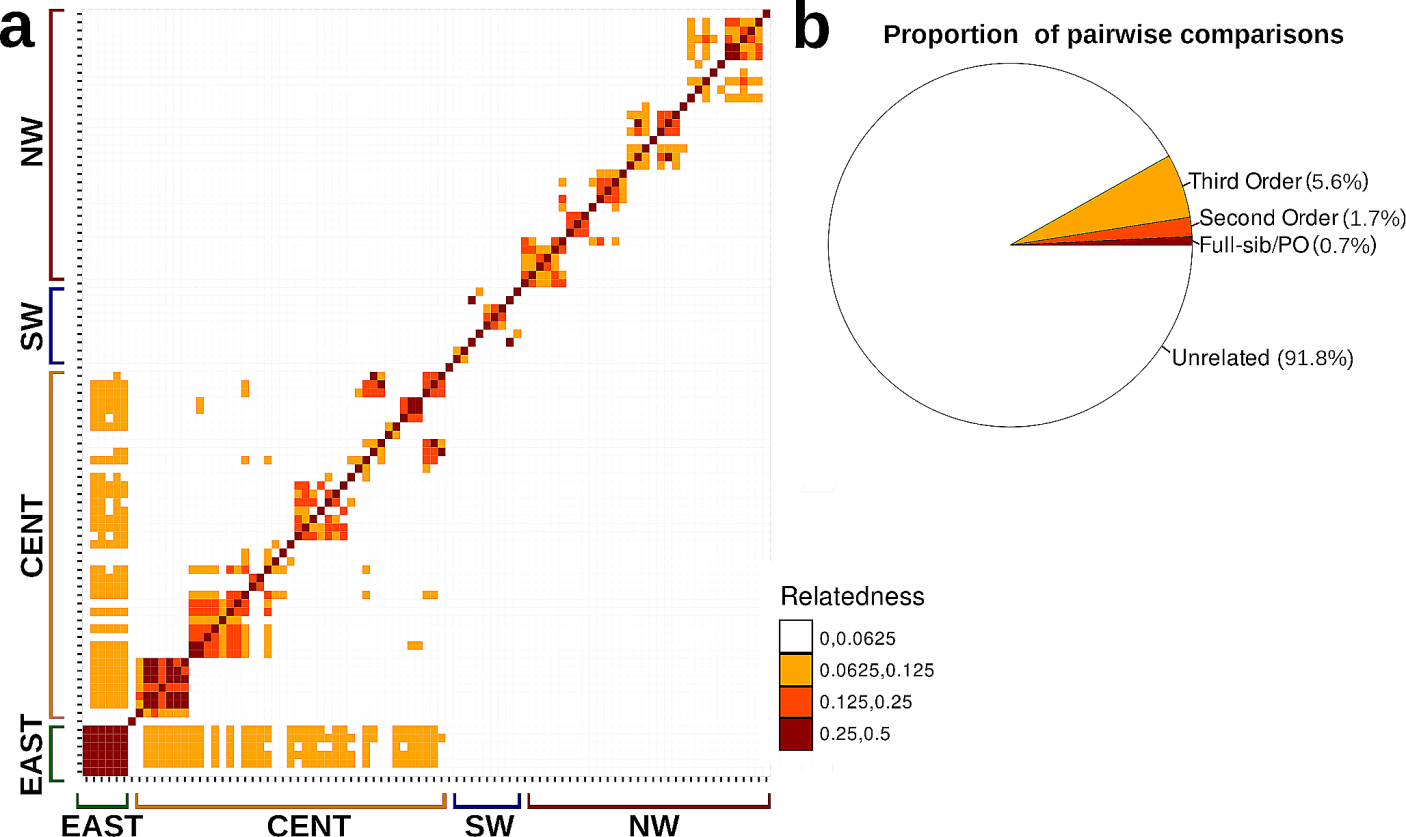
Heatmap of the paired relatedness values of *Cucurbita radicans* in Central Mexico. The pie chart represents the proportion of individuals assigned to each relatedness class. Color scales represent the value of relatedness

The relatedness test showed that most of the paired comparisons corresponded to unrelated individuals (91.8%), although the highly related individuals were within localities (Fig. 2). Interestingly, the MESA I and II localities showed low, but significant values of relatedness with almost all individuals (mean value *r* = 0.05), however this may be explained by the low genetic diversity of MESA localities.

Pairwise *F*_*ST*_ values among localities displayed values from 0.004 for the nearest localities (MESA I vs. MESA II, not significant) to 0.332 (MESA II vs. SAGU) (Fig. S1, Table S3). Although the highest *F*_*ST*_ values were not between the furthest localities, we observed a regional structure, dividing the samples into at least three main genetic groups, suggesting a geographic component influencing genetic structure (but see results below).

**Fig. 3:**
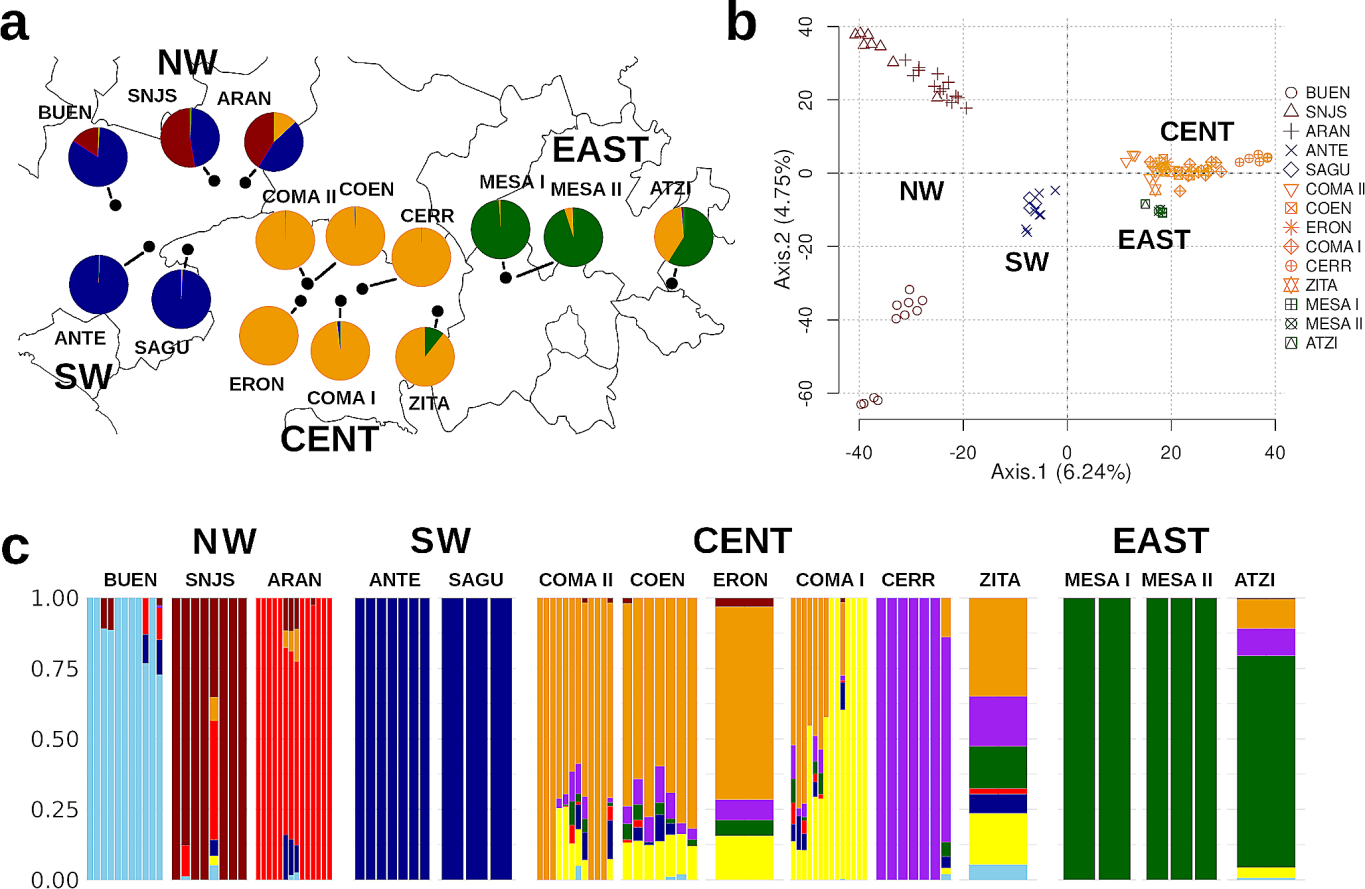
**a**) Geographic distribution of admixture values for *K* = 4 in *Cucurbita radicans* in Central Mexico obtained with conStruct. The assigned genetic clusters are NW, SW, CENT, and EAST. Colors in pie charts represent the genetic admixture values per locality. **b**) PCA at the individual level, symbols correspond to each locality, and colors correspond to each genetic group. **c**) Admixture results for *K* = 8, localities are ordered by longitude and grouped according to the genetic group

The conStruct analysis indicated an optimal CV value at *K* = 4 for both the spatial and non-spatial models. Moreover, we did not find statistical differences between models (*P* = 0.4301, Welch two-sample test), while the admixture contribution along *K* values showed a contribution decay after *K* = 3 in the spatial model and *K* = 4 for non-spatial. We kept the non-spatial model, as its value of *K* = 4 was consistent in both tests, however, results were consistent between models (Fig. S2). With these results, we identified four main genetic groups with unique genetic components concordant with their distribution in the North-West, South-West, Center, and East (hereafter NW, SW, CENT, and EAST) in the geographic range of the samples (Fig. 3a).

The PCA displayed a consistent grouping with conStruct, with the difference that the locality BUEN was separated from SNJS and ARAN (Fig. 3b). On the other hand, the admixture analyses showed that on average, the lower value of CV was *K* = 8 with a range of *K* = 7 to *K* = 9 (Fig. 3c, Fig. S3). Nevertheless, it is possible to distinguish the four groups with additional structure within each one, showing highly admixed individuals (in particular in the CENT genetic cluster) within and among localities, but to a lesser extent among genetic groups defined with conStruct and PCA. Even though we found a general agreement in the structure patterns (including the *F*_*ST*_ clustering), the discrepancies among methods reflect the complex genetic structure of this species.

Using the identified grouping, we observed a range of *H*_*E*_ = 0.176 to 0.273, being the EAST, the least diverse group, with a deficiency of homozygous individuals *F*_*IS*_ of − 0.615 (Table 1). The pairwise *F*_*ST*_ showed significant values, ranging from 0.072 (CENT and SW) to 0.218 (SW and EAST), with the EAST cluster as the most differentiated according to the geographic distance. Furthermore, both migration analyses (BayesASS and migrate-n) showed low non-significant values of gene flow among genetic groups, suggesting that the genetic divergence among genetic groups is due to genetic drift, with null or very restricted gene flow among them, current or historical (see rCCR below).

**Table 1:** Summary statistics of the geographic-genetic groups in *Cucurbita radicans* from Central Mexico. Standard deviation in parenthesis

Genetic cluster	H_E_	H_O_	F_IS_	*N*
NW	0.2420 (0.1598)	0.2220 (0.2220)	−0.0140 (0.2004)	33
SW	0.2457 (0.1858)	0.2370 (0.2370)	0.0203 (0.2982)	10
CENT	0.2735 (0.1414)	0.2766 (0.1504)	0.0140 (0.1609)	33
EAST	0.1705 (0.2244)	0.3080 (0.4212)	−0.615 (0.4530)	6

The environmental variables displayed the most extreme differences between the geographically distant NW and EAST genetic groups, while SW and CENT clusters had intermediate values (Fig. S4). Overall, the EAST group had the most divergent conditions, in particular in Bio.14 and Bio.15 (precipitation of driest month, and precipitation seasonality). It is noteworthy that despite the geographic closeness of NW and SW groups, their localities were environmentally very different.

**Fig. 4:**
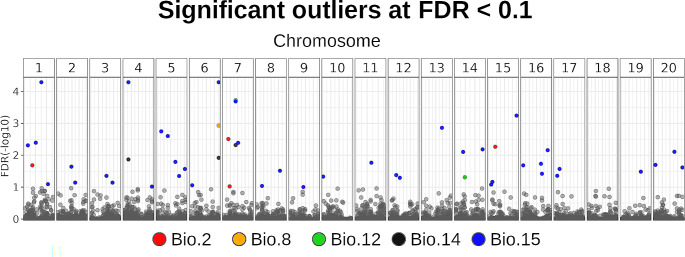
Manhattan plot showing the outlier loci obtained with latent factor mixed model (LFMM) analysis of the R package LEA for *Cucurbita radicans* from Central Mexico. On y-axis the False Discovery Rate (FDR) values are presented. Colored points correspond to significant outliers, each color corresponds to associated environmental variables. Loci are grouped by chromosome and position according to the *Cucurbita maxima* genome

As mentioned above, the LEA detection test identified 47 outlier-loci (Fig. 4). Most of them (80.7%) were associated with variable Bio.15, while 7.6% to Bio.2, 6% to Bio.14, and 3.8% and 1.9% to Bio.12 and Bio.8, respectively. Moreover, two loci (Chr6.loc30466 and Chr.7loc31868) were associated with three climatic variables, one locus (Chr4.loc14947) with two climatic variables and the rest with only one. The gene-ontology (GO) search indicated that most outliers related to “Molecular-associated function”, and the most represented GO was “Integral component of membrane function” (Fig. S5a, b). Among the biological putative functions, we observed response to stress, immune response, regulation of gene expression, and transcription processes (Fig. S5b, Table S5). Also, from the 47 outliers, eleven loci (23%) had no hit or an e-value beyond the threshold.

**Fig. 5:**
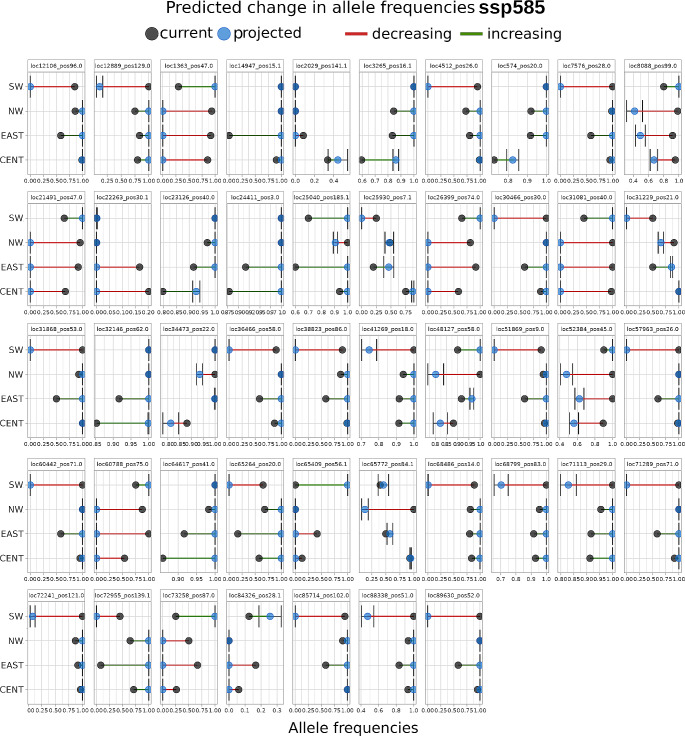
Outlier loci predicted frequencies obtained with AlleleShift in *Cucurbita radicans* in Central Mexico using the ssp585 model projected over the year 2100

Allele-shift analysis indicated contrasting behaviors among outliers within and between groups (Fig. 5). The results for the ssp585 scenario predicted an increase in frequency on 51.5% of the outlier loci, a decrease in 33.5%, while 15% of these loci showed no change. Notably, the SW group displayed the most contrasting trends, as it was the cluster that had the most loci without change, fewer loci at frequency increase, and more at decrease in the predicted frequencies (Fig. 5).

**Fig. 6:**
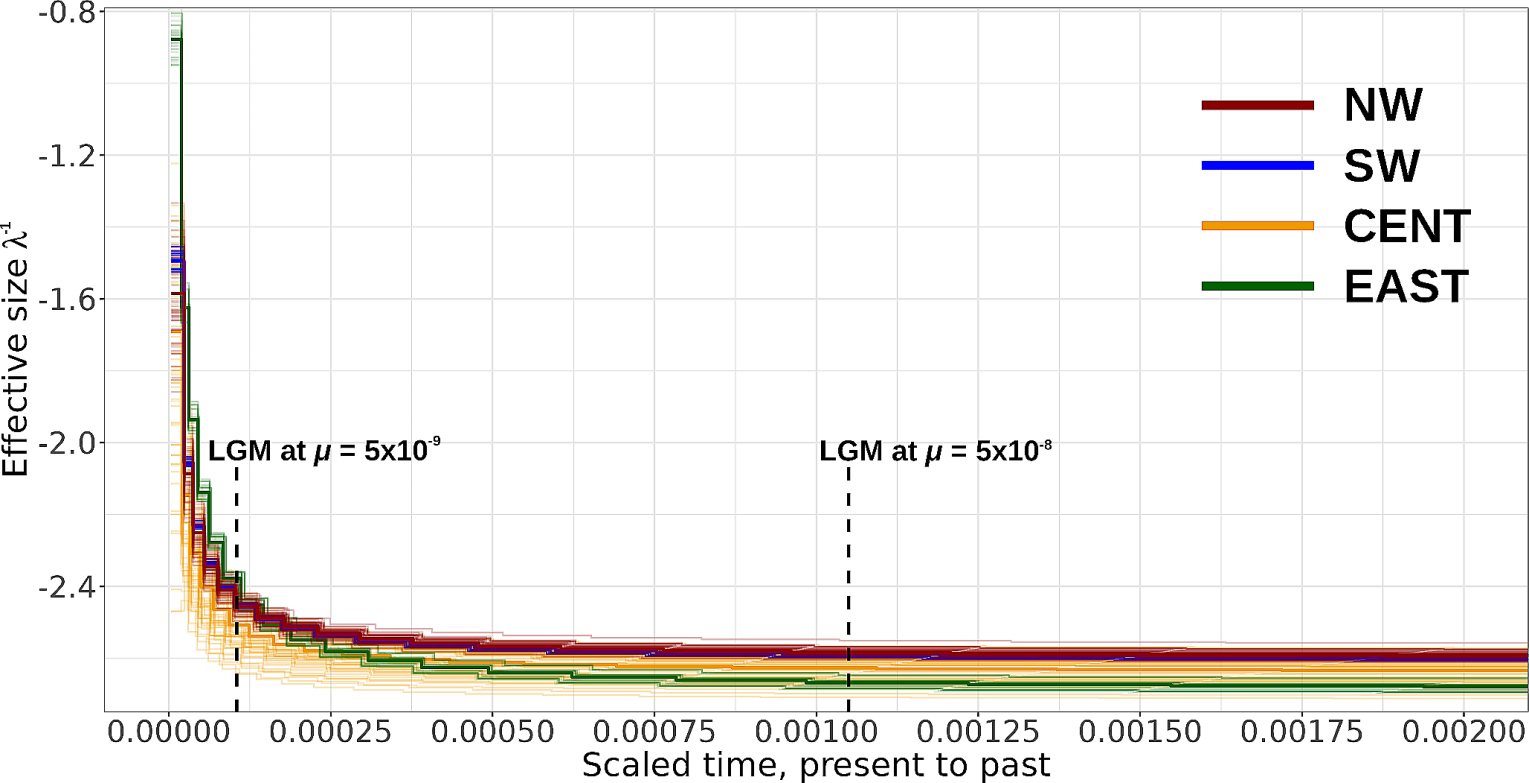
Demographic analyses obtained with MSMC2 in *Cucurbita radicans* from Central Mexico. The x-axis presents the scaled time from present to past as *t / (µg)* where *µ* is the mutation rate and *g* is the generation time. The y-axis, presents the scaled effective size in time interval *i* as λ^−1^ = *N*_*i*_*/ N*_*0*_ where *N* is *θ / 4µ*. Each line color represents the genetic groups previously identified. The graph shows the position of the Last Glacial Maximum (21,000 years in the past) according to the proposed mutation range (5 × 10^− 8^ to 5 × 10^− 9^)

The MSMC2 demographic analyses showed that all genetic groups had a historical steady size, followed by a sudden demographic expansion, with similar trends in the four genetic groups (Fig. 6). Moreover, with the proposed mutation rate range (5 × 10^−8^ to 5 × 10^−9^), we obtained recent dates for the population growth during or shortly after the LGM (∼ 21,000 years ago). On the other hand, the ratio of the cross coalescence rate (rCCR) analysis gave results close to 0 for either of the group pairings, so it was not possible to approximate the divergence date between genetic groups, suggesting an ancient divergence date predating the coalescent times of sampled haplotypes.

## Discussion

In this paper, we evaluated the genomic diversity of *C. radicans*, a relatively uncommon wild cucurbit from Central Mexico, and explored the evolutionary factors influencing its genetic variation. Even though it was considered an abundant species as reported by Lira (2001), in less than 20 years the species became scarce, due principally to habitat loss and population fragmentation (Aragón et al. 2020). Our field observations confirmed that *C. radicans* is currently a rare species in most of its distribution with fragmented populations. Nevertheless, *C. radicans* exhibited relatively high levels of genetic diversity (*H*_E_ = 0.268) in comparison to studies with SNPs in other species from the genus. For example, *C. moschata* showed *H*_E_ values from 0.202 (*N* = 63, 224 SNPs; Nguyen et al. 2020) to 0.327 (*N* = 104, 2071 SNPs; Lee et al. 2020) with high levels of inbreeding, while *C. maxima* displayed an *H*_E_ = 0.27 (*N* = 73, 224 SNPs; Nguyen et al. 2020)d *pepo* a *H*_*E*_ = 0.18 (*N* = 45, 224 SNPs; Nguyen et al. 2020) in samples from accessions in germplasm collections. Further, in samples from Mexico, Martínez-González et al. (2021) reported values of *H*_E_ = 0.196 (*N* = 64 2,016 SNPs) in Mexican landraces, while Aguirre-Dugua et al. (2023) *H*_*E*_ = 0.226 for *C. ficifolia* (*N* = 36, 2,524 SNPs). However, it is important to note that these studies focused on crops and accessions from collections, with smaller sample sizes and fewer analyzed SNPs compared to our study. Furthermore, it is expected that the wild relatives of crops have higher genetic diversity (Zhang et al. 2017). In the case of *C. radicans*, our estimates of diversity encompass genetic structure from divergent lineages adapted to a variety of environments and locations, which contributed to relatively high genetic diversity. To assess, and objectively compare the levels of genetic diversity of *C. radicans*, it is necessary to estimate the population genetic diversity of other wild pumpkins species.

Regarding the spatial distribution of genetic diversity, we found intricate genetic structure patterns, partly influenced by local differentiation, local adaptation, and limited dispersal. Indeed, we found higher genetic relatedness for individuals from the same or close localities. In particular, we detected identical genotypes shared by the MESA I and MESA II localities, but the samples were collected more than 300 m apart, so we consider it highly unlikely that fruits came from the same plant. Although this may suggest a certain degree of clonality, it is more feasible that the seemingly identical genotypes were the product of the dispersion of close relatives and low genomic diversity due to small populations sizes in these localities, in addition to stringent filters applied for the data set.

Overall, we found that genetic structure resulted from geographic isolation and differences in local environmental conditions. Although the results displayed low genetic structure at a local scale, we observed localities with low differentiation at relatively long distances, and conversely, localities with high structure at close distances. For instance, COMA I and COMA II showed a *F*_ST_ = 0.061 at a distance of 38.7 km between them, while SNJS vs. ARAN had a *F*_*ST*_ = 0.133 at 31.43 km between them. Furthermore, conStruct did not find statistical differences between the spatial and non-spatial models. This suggests that, aside from the geographic distribution, external factors like the environment may be influencing the patterns of genetic differentiation.

A previous study established that *C. radicans* separated from group *C. pedatifolia*-*C. foetidissima* around 6.5 mya (Castellanos-Morales et al. 2018) during a period of active volcanism at the Trans-Mexican Volcanic Belt (Gómez-Tuena et al. 2007). The formation of the Trans-Mexican Volcanic Belt created different habitats and barriers to dispersal that promoted the divergence of natural populations (Bryson et al. 2011; Mastretta-Yanes et al. 2015), leading to fragmented and mosaic-like distribution of *Cucurbita* species (Castellanos-Morales et al. 2018). Accordingly, our results of BayesAss, migrate-n and the rCCR analysis suggested that the genetic groups of *C. radicans* may have been genetically isolated for a long time, as the time inference of the coalescent-based methods is limited to the maximum coalescence time of the sample, approximately 4*Ne* generations (Kingman 1982). On the other hand, our demographic analysis showed a historically steady population size followed by rapid growth during the LGM, consistent with the historical distribution models proposed by Castellanos-Morales et al. (2018) (see Fig. S6). Under these hypotheses, the range expansion should be coupled with a long-range dispersion of fruits by the Pleistocene megafauna (Kistler et al. 2015). Then, after the extinction of the Pleistocene North American megafauna during the Younger Dryas (Alley 2000) 13,800 to 11,400 years ago (Faith and Surovell 2009), the wild pumpkin populations remained genetically isolated. Also, previous studies pointed out that small mammals are not good dispersers of *Cucurbita* seeds due to the unpalatability of the cucurbitacin produced by the plants (Kistler et al. 2015). If this hypothesis is viable, similar patterns of genetic structure should be found in populations of other wild pumpkin species like *C. foetidissima*, *C. pedatifolia* and *C. scabridifolia*.

On the other hand, our demographic analysis showed a small but constant historical population size followed by rapid growth during the LGM, consistent with the historical distribution models proposed by Castellanos-Morales et al. (2018) (Fig. S6). The rapid demographic growth associated with range expansion is expected to promote gene flow among populations. However, the genetic structure detected suggests that the demographic growth in *C. radicans* favored gene flow within the range of genetic groups but maintaining the isolation among them.

It has been well documented that the historical climate changes during the Pleistocene glaciations significantly influenced the evolution of vegetation in Mexico (Rzedowski, 1978), leaving different footprints in the genetic diversity of plant populations (Jaramillo-Correa et al. 2009; Ornelas et al. 2013). For example, species distributed in Central Mexico like *Dioon edule* (Zamiaceae) suffered a population density reduction during glaciation followed by range expansion after the glaciers retracted (González-Astorga et al. 2003), while populations of *Agave kerchovei* (Asparagaceae) went through a range expansion during the LGM, but contracted during the Mid-Holocene, and have remained stable since (Aguirre-Planter et al. 2020).

We identified outlier loci associated with environmental variables, mostly with precipitation, and predicted frequency changes in the future under the climate change models. Also, while it is difficult to relate specific loci to an actual response to an environmental variable based solely on allele frequencies (Kawecki and Ebert 2004), our findings suggest that, in addition to the differentiation due to genetic drift, there is evidence of the role of the environment shaping genetic diversity. It is worth mentioning that our AlleleShift analysis projected the allele frequencies using the current distribution records. This assumes that the species will remain in the current distribution area or close to it. As previously mentioned, the natural dispersers of *Cucurbita* fruits went extinct during the Holocene, and even though the fruits may potentially be dispersed by livestock, their dispersal range is limited to areas with farms, and it is unlikely to extend beyond the current range. Moreover, the environmental changes under global climate change occured rapidly, taking about 100 years. In this context, the AlleleShift results show the importance of maintaining genetic diversity, as some variants will become fixed according to the models for the *insitu* survival of *C. radicans* populations.

Contrary to our first prediction, in *C. radicans* the genetic groups displayed moderate to high genetic diversity, except the EAST group, where all localities showed a scarcity of individuals. In particular, the *F*_*IS*_ in this genetic group indicated an excess of heterozygotes (− 0.933 and − 0.925, Table S2) that may suggest a very recent population bottleneck (Luikart and Cornuet 1998). However, the moderate-high levels of genetic diversity in the overall sample may be the result of a long period of constant size of the genetic groups along the genetic isolation between them before the Mid-Holocene. Furthermore, the demographic growth let the populations expand their range, which in turn aided in maintaining diversity (fourth prediction). However, our results indicate that human activity over the past 20 years has affected the genetic diversity at the local level, so it is important for the isolated localities to connect with other localities to maintain genetic diversity.

We found genetic structure at local and regional scale influenced by geographic distribution and environmental variations with a notable response of the outlier loci to future climate change according to the second and third predictions. Even though the results suggest loci under selective pressures, local adaptation should be corroborated with analyses such as common garden experiments, where genotypes should perform better in their native environmental conditions (Kawecki and Ebert 2004).

Finally, while it is difficult to establish priority areas for conservation in a species such as *C. radicans* with high genetic structure and contrasting environmental conditions, according to our criteria (representation of genetic and environmental variation in PCAs, representation of all genetic components obtained in genotype assignment analysis, and at least a 90% of polymorphic SNPs), we propose the following localities as the areas of major conservation interest from a genetic, historical and future relevance perspective: BUEN, SNJS, SAGU COMA I, CERR, ZITA, and ATZI. With the preservation of these localities, 99% of loci will be polymorphic, a value of *H*_*E*_ = 0.2639 will be preserved, and most of the genetic and environmental variation is represented. Finally, although our sampling covered most of the geographical range of *C. radicans*, we were unable to collect the populations at the lower altitudes (less than 1000 m.a.s.l). According to WorldClim data, these populations have on average warmer and slight wetter conditions (mean annual temperature 21.75 °C, annual precipitation 877 mm) compared to the populations included in the present study (mean annual temperature 17.05 °C, annual precipitation 861 mm). Given their geographical distribution (around 20.3 N, −101.5 W), and according to our results, it is possible that these populations constitute a distinct genetic cluster, with potentially unique genetic variants associated with their specific environmental conditions, and it is possible that outlier loci may be detected from these populations. In that case, this hypothetical genetic cluster may be relevant for conservation, and more effort should be made to sample these populations and evaluate their genetic diversity. It is worth noting however, that the geographical records for those populations predate 1994 (GBIF, www.gbif.org/; CONABIO, enciclovida.mx/).

## Conclusions

*Cucurbita radicans* is a species with relatively high levels of genetic differentiation adapted to wide environmental conditions. According to previous studies in the area, the history of the species, including environmental shifts, geological events, and the consequent population size changes, our results indicate that the preservation of its genetic diversity may be crucial for the survival of the species under a climate change scenario. The diversity of environments and the intrinsic characteristics of the species, make *C. radicans* an interesting model for the search of genetic variants for crop improvement.

## Electronic supplementary material

Below is the link to the electronic supplementary material.

Supplementary Material 1

Supplementary Material 2

## Data Availability

Raw read data are available at NCBI GenBank Bioproject number PRJNA976254 (accession numbers SRR24737162-SRR24737255).

## Declarations

### Field study permissions

Samplings were conducted under the permit by the Secretaría de Medio Ambiente y Recursos Naturales, Subsecretaría de Gestión para la Protección Ambiental, Dirección General de Gestión Forestal y de Suelos, issued by the Mexican government (SGPA/DGGFS/712/1596/17).
