# Supplementary material for: Conservation genomics of the wild pumpkin Cucurbita radicans in Central Mexico: The influence of a changing environment on the genetic diversity and differentiation of a rare species
Source: J Plant Res. 2024 Jul 8;137(5):799–813. doi: 10.1007/s10265-024-01552-1 (PMC11393293; doi:10.1007/s10265-024-01552-1)
Supplement: Supplementary file 1 — Supplementary Material 1 [file 10265_2024_1552_MOESM1_ESM.pdf]

Conservation genomics of the wild pumpkin *Cucurbita radicans* in Central Mexico: The influence of a changing environment on the genetic diversity and differentiation of a rare species

Journal of Plant Research

Jaime Gasca-Pineda[1,2*],Brenda Monterrubio[2] , Guillermo Sánchez-de la Vega[1] , Erika Aguirre-Planter[1] , Rafael Lira-Saade[2] , Luis E. Eguiarte[1*].

[1] Departamento de Ecología Evolutiva, Instituto de Ecología, Universidad Nacional Autónoma de México. Circuito Exterior s/n Anexo al Jardín Botánico, 04510 Ciudad de México, México.
[2] Unidad de Biotecnología y Prototipos, Facultad de Estudios Superiores Iztacala, Universidad Nacional Autónoma de México. Av. De Los Barrios 1, Col. Los Reyes Iztacala, 54090, Tlalnepantla, Estado de México, México.
*corresponding authors fruns@unam.mx, jaimegasca@yahoo.com

Supplemental Figures

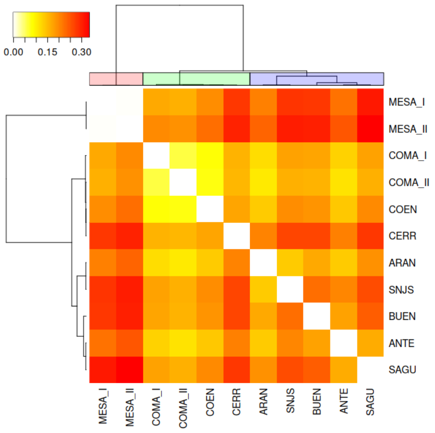

Figure S1. Paired F_{ST} among localities of *Cucurbita radicans*. Distance dendrogram was created using the complete linkage method. Colored boxes indicate groups identified by the broken-stick method.

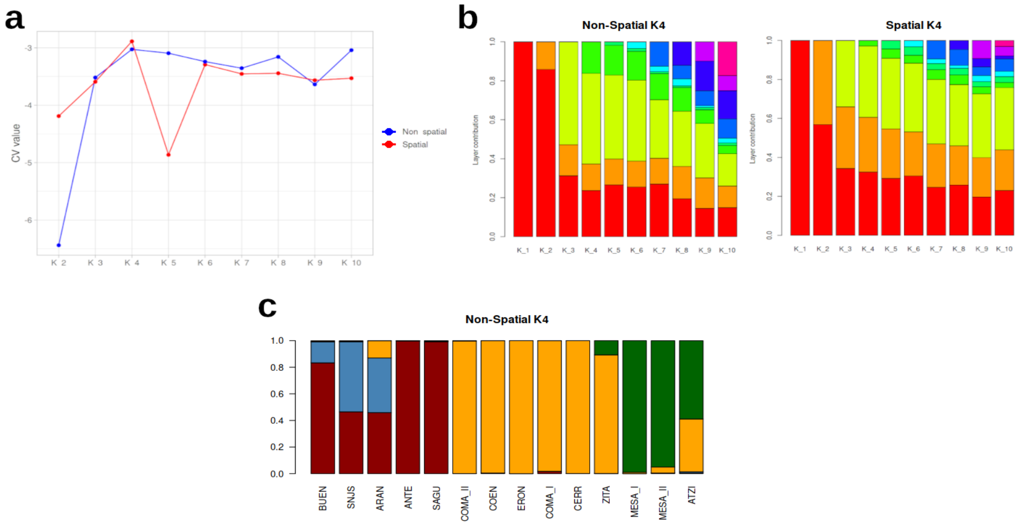

Figure S2. a) Cross-validation analysis for the optimal *K* in *Cucurbita radicans* from Central Mexico for the non-Spatial (blue) and Spatial (red). b) Admixture contribution for non-Spatial models and Spatial. c) Barplot of the median values of admixture for 10 independent runs for *K* = 4.

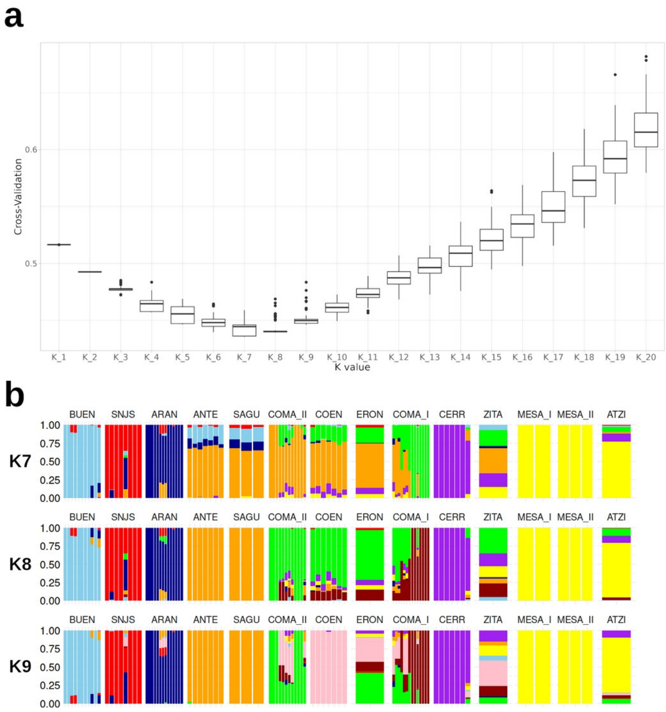

Figure S3. a) Cross-validation tests for the optimal value of *K* for 100 independent runs. b) Barplots of the admixture values from *K* = 7 to *K* = 9.

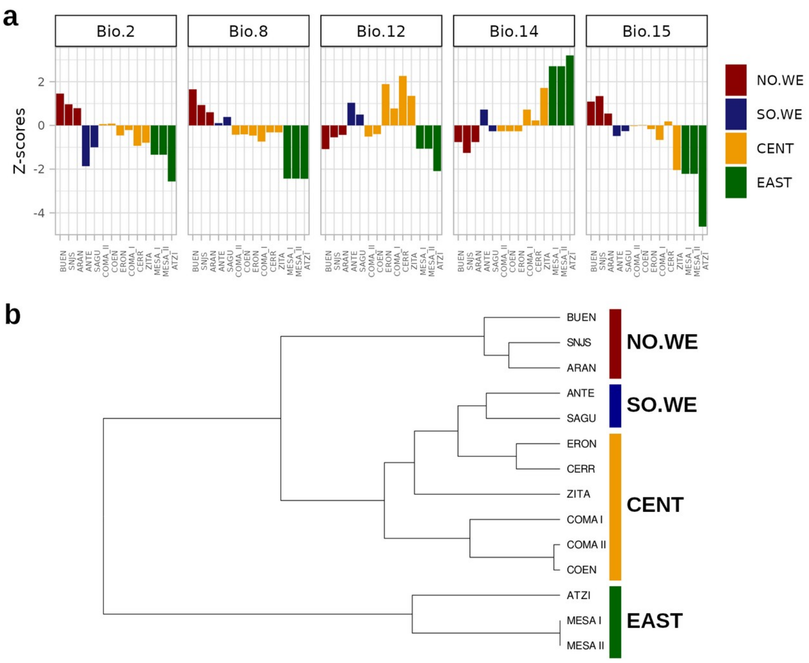

Figure S4. a) Z-scores values of the bio-variables per locality, color represents the genetic group. b) Dendrogram of the Euclidean distances for the first two components of a PCA of the environmental

variables (93.2 % of cumulative variance). Color represents the genetic groups detected in this study.

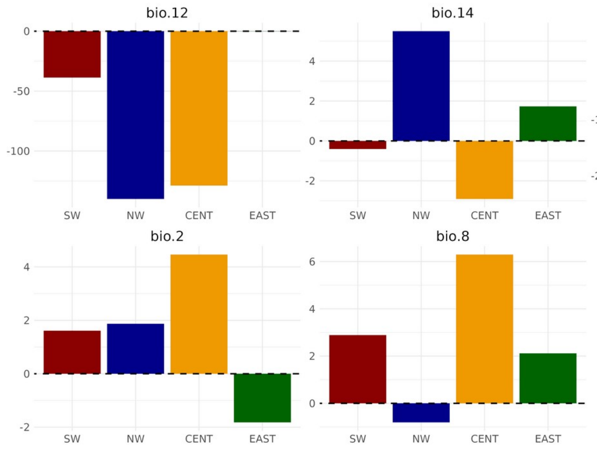

Figure S4. Change of the bioclimatic variables analyzed in this study from the current conditions to 100 years to the future (ssp585 model), for the genetic groups. The dashed line corresponds to zero and represent no differences between periods of time.

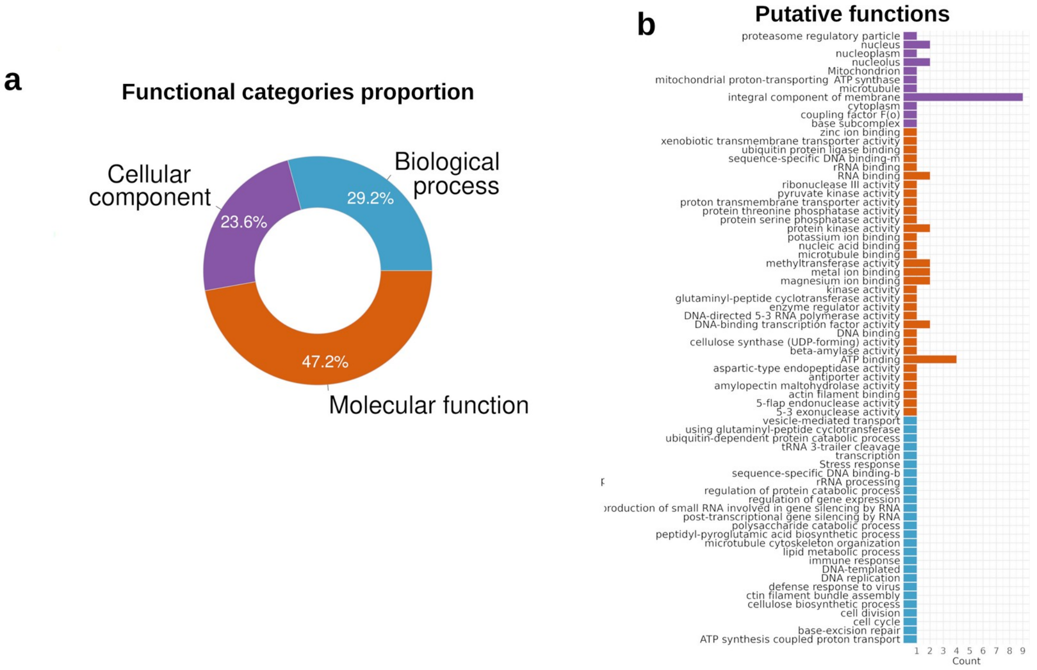

Figure S5. a) Proportion of functional categories found for outlier loci according to the UniProt database. b) Putative function assigned from the gene-ontology match according to the UniProt database

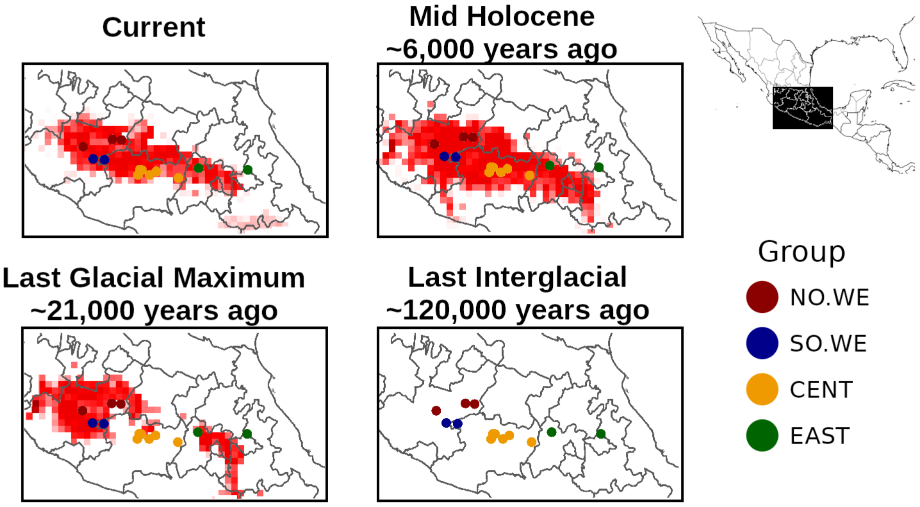

Figure S6. Species Distribution Models for *Cucurbita radicans* for the Last Inter Glacial, Last Glacial Maximum, Mid-Holocene and Present. The color dots show de distribution of the localities and their genetic group. The models were provided by the authors of Castellanos et al. 2018.
