# Supplementary material for: Conservation genomics of the wild pumpkin Cucurbita radicans in Central Mexico: The influence of a changing environment on the genetic diversity and differentiation of a rare species
Source: J Plant Res. 2024 Jul 8;137(5):799–813. doi: 10.1007/s10265-024-01552-1 (PMC11393293; doi:10.1007/s10265-024-01552-1)
Supplement: Supplementary file 2 — Supplementary Material 2 [file 10265_2024_1552_MOESM2_ESM.pdf]

Conservation genomics of the wild pumpkin *Cucurbita radicans* in Central Mexico: The influence of a changing environment on the genetic diversity and differentiation of a rare species

Journal of Plant Research

Jaime Gasca-Pineda[1,2*],Brenda Monterrubio[2] , Guillermo Sánchez-de la Vega[1] , Erika Aguirre-Planter[1] , Rafael Lira-Saade[2] , Luis E. Eguiarte[1*].

[1] Departamento de Ecología Evolutiva, Instituto de Ecología, Universidad Nacional Autónoma de México. Circuito Exterior s/n Anexo al Jardín Botánico, 04510 Ciudad de México, México.
[2] Unidad de Biotecnología y Prototipos, Facultad de Estudios Superiores Iztacala, Universidad Nacional Autónoma de México. Av. De Los Barrios 1, Col. Los Reyes Iztacala, 54090, Tlalnepantla, Estado de México, México.
*corresponding authors fruns@unam.mx, jaimegasca@yahoo.com

Table S1. Information of the *Cucurbita radicans* samples analyzed in this study. Fastq name corresponds to the sample accessions in GenBank (SRR24737162-SRR24737255).

Fastq name	Individual key	Locality key	Name of the locality	State	Longitude	Latitude
4542_1-1b_S454001	COMA_II_1-1b	COMA_II	Comanja, Municipio de Coeneo	Michoacán	-101.6661279	19.747
4542_1-1c_S417001	COMA_II_1-1c	COMA_II	Comanja, Municipio de Coeneo	Michoacán	-101.6661279	19.747
4542_1-2b_S392001	COMA_II_1-2b	COMA_II	Comanja, Municipio de Coeneo	Michoacán	-101.6661279	19.747
4542_12_S12001	COMA_II_12	COMA_II	Comanja, Municipio de Coeneo	Michoacán	-101.6661279	19.747
4542_12a_S384001	COMA_II_12a	COMA_II	Comanja, Municipio de Coeneo	Michoacán	-101.6661279	19.747
4542_12b_S436001	COMA_II_12b	COMA_II	Comanja, Municipio de Coeneo	Michoacán	-101.6661279	19.747
4542_14a_S288001	COMA_II_14a	COMA_II	Comanja, Municipio de Coeneo	Michoacán	-101.6661279	19.747
4542_14b_S25001	COMA_II_14b	COMA_II	Comanja, Municipio de Coeneo	Michoacán	-101.6661279	19.747
4542_1a_S385001	COMA_II_1a	COMA_II	Comanja, Municipio de Coeneo	Michoacán	-101.6661279	19.747
4542_1b_S300001	COMA_II_1b	COMA_II	Comanja, Municipio de Coeneo	Michoacán	-101.6661279	19.747
4542_2b_S347001	COMA_II_2b	COMA_II	Comanja, Municipio de Coeneo	Michoacán	-101.6661279	19.747
4542_2c_S371001	COMA_II_2c	COMA_II	Comanja, Municipio de Coeneo	Michoacán	-101.6661279	19.747
Ante_1-1a_S401001	ANTE_1-1a	ANTE	Antena, Municipio de Teocuitatlán de Corona	Jalisco	-103.20194444	20.0823889
Ante_2-2a_S396001	ANTE_2-2a	ANTE	Antena, Municipio de Teocuitatlán de Corona	Jalisco	-103.20194444	20.0823889

Ante_3-2a_S472001	ANTE_3-2a	ANTE	Antena, Municipio de Teocuitatlán de Corona	Jalisco	-103.20194444	20.0823889
Ante_3-3a_S312001	ANTE_3-3a	ANTE	Antena, Municipio de Teocuitatlán de Corona	Jalisco	-103.20194444	20.0823889
Ante_3-3b_S337001	ANTE_3-3b	ANTE	Antena, Municipio de Teocuitatlán de Corona	Jalisco	-103.20194444	20.0823889
Antena_1-1b_S83001	ANTE_1-1b	ANTE	Antena, Municipio de Teocuitatlán de Corona	Jalisco	-103.20194444	20.0823889
Antena_2-2b_S65001	ANTE_2-2b	ANTE	Antena, Municipio de Teocuitatlán de Corona	Jalisco	-103.20194444	20.0823889
Atzi_2_S346001	ATZI_2	ATZI	Atzintlimeya, San José Atzintlimeya, Municipio de Chignahuapan	Puebla	-98.11913889	19.74377778
Buen_11a_S467001	BUEN_11a	BUEN	Buenavista, Municipio de Tlajomulco de Zúñiga	Jalisco	-103.529465	20.459774
Buen_11b_S383001	BUEN_11b	BUEN	Buenavista, Municipio de Tlajomulco de Zúñiga	Jalisco	-103.529465	20.459774
Buen_13a_S389001	BUEN_13a	BUEN	Buenavista, Municipio de Tlajomulco de Zúñiga	Jalisco	-103.529465	20.459774
Buen_13b_S459001	BUEN_13b	BUEN	Buenavista, Municipio de Tlajomulco de Zúñiga	Jalisco	-103.529465	20.459774
Buen_1a_S416001	BUEN_1a	BUEN	Buenavista, Municipio de Tlajomulco de Zúñiga	Jalisco	-103.529465	20.459774
Buen_1b_S404001	BUEN_1b	BUEN	Buenavista, Municipio de Tlajomulco de Zúñiga	Jalisco	-103.529465	20.459774
Buen_2a_S426001	BUEN_2a	BUEN	Buenavista, Municipio de Tlajomulco de Zúñiga	Jalisco	-103.529465	20.459774
Buen_2b_S411001	BUEN_2b	BUEN	Buenavista, Municipio de Tlajomulco de Zúñiga	Jalisco	-103.529465	20.459774
Buen_4a_S375001	BUEN_4a	BUEN	Buenavista, Municipio de Tlajomulco de Zúñiga	Jalisco	-103.529465	20.459774
Buen_6a_S364001	BUEN_6a	BUEN	Buenavista, Municipio de Tlajomulco de Zúñiga	Jalisco	-103.529465	20.459774
Buen_8a_S297001	BUEN_8a	BUEN	Buenavista, Municipio de Tlajomulco de Zúñiga	Jalisco	-103.529465	20.459774
Coma_12_S415001	COMA_I_12	COMA_I	Comanja, Cuanajillo del Toro, Municipio de Morelia	Michoacán	-101.33789063	19.58670403
Coma_20a_S462001	COMA_I_20a	COMA_I	Comanja, Cuanajillo del Toro, Municipio de Morelia	Michoacán	-101.33789063	19.58670403
Coma_20b_S299001	COMA_I_20b	COMA_I	Comanja, Cuanajillo del Toro, Municipio de Morelia	Michoacán	-101.33789063	19.58670403

Coma_20c_S289001	COMA_I_20c	COMA_I	Comanja, Cuanajillo del Toro, Municipio de Morelia	Michoacán	-101.33789063	19.58670403
Coma_23_S386001	COMA_I_23	COMA_I	Comanja, Cuanajillo del Toro, Municipio de Morelia	Michoacán	-101.33789063	19.58670403
Coma_23b_S362001	COMA_I_23b	COMA_I	Comanja, Cuanajillo del Toro, Municipio de Morelia	Michoacán	-101.33789063	19.58670403
Coma_2a_S313001	COMA_I_2a	COMA_I	Comanja, Cuanajillo del Toro, Municipio de Morelia	Michoacán	-101.33789063	19.58670403
Coma_5a_S457001	COMA_I_5a	COMA_I	Comanja, Cuanajillo del Toro, Municipio de Morelia	Michoacán	-101.33789063	19.58670403
Coma_5b_S390001	COMA_I_5b	COMA_I	Comanja, Cuanajillo del Toro, Municipio de Morelia	Michoacán	-101.33789063	19.58670403
Coma_8-1c_S427001	COMA_I_8-1c	COMA_I	Comanja, Cuanajillo del Toro, Municipio de Morelia	Michoacán	-101.33789063	19.58670403
Coma_8-2a_S413001	COMA_I_8-2a	COMA_I	Comanja, Cuanajillo del Toro, Municipio de Morelia	Michoacán	-101.33789063	19.58670403
Coma_8-2b_S433001	COMA_I_8-2b	COMA_I	Comanja, Cuanajillo del Toro, Municipio de Morelia	Michoacán	-101.33789063	19.58670403
Coma_8a_S351001	COMA_I_8a	COMA_I	Comanja, Cuanajillo del Toro, Municipio de Morelia	Michoacán	-101.33789063	19.58670403
Coma_8b_S379001	COMA_I_8b	COMA_I	Comanja, Cuanajillo del Toro, Municipio de Morelia	Michoacán	-101.33789063	19.58670403
Cons_1-2a_S315001	COEN_1-2a	COEN	Coeneo, Municipio de Coeneo	Michoacán	-101.65913888	19.7404444
Cons_1-6a_S350001	COEN_1-6a	COEN	Coeneo, Municipio de Coeneo	Michoacán	-101.65913888	19.7404444
Cons_1-6b_S314001	COEN_1-6b	COEN	Coeneo, Municipio de Coeneo	Michoacán	-101.65913888	19.7404444
Const_1-2b_S293001	COEN_1-2b	COEN	Coeneo, Municipio de Coeneo	Michoacán	-101.65913888	19.7404444
Const_1-8a_S5001	COEN_1-8a	COEN	Coeneo, Municipio de Coeneo	Michoacán	-101.65913888	19.7404444
Const_2c_S442001	COEN_2c	COEN	Coeneo, Municipio de Coeneo	Michoacán	-101.65913888	19.7404444
Const_6c_S388001	COEN_6c	COEN	Coeneo, Municipio de	Michoacán	-101.65913888	19.7404444

			Coeneo			
Eron_57_S424001	ERON_57	ERON	Erongarícuaro, Municipio de Erongarícuaro	Michoacán	-101.727543	19.585783
Golf_1-3a_S15001	CERR_1-3a	CERR	Cerro Punhuato, Municipio de Morelia	Michoacán	-101.12907	19.69551
Golf_1-3b_S355001	CERR_1-3b	CERR	Cerro Punhuato, Municipio de Morelia	Michoacán	-101.12907	19.69551
Golf_2-1a_S308001	CERR_2-1a	CERR	Cerro Punhuato, Municipio de Morelia	Michoacán	-101.12907	19.69551
Golf_2-1b_S398001	CERR_2-1b	CERR	Cerro Punhuato, Municipio de Morelia	Michoacán	-101.12907	19.69551
Golf_2-1c_S379001	CERR_2-1c	CERR	Cerro Punhuato, Municipio de Morelia	Michoacán	-101.12907	19.69551
Golf_2-4a_S449001	CERR_2-4a	CERR	Cerro Punhuato, Municipio de Morelia	Michoacán	-101.12907	19.69551
Golf_3-2_S330001	CERR_3-2	CERR	Cerro Punhuato, Municipio de Morelia	Michoacán	-101.12907	19.69551
Mes1_2_S358001	MESA_I_2	MESA_I	La Mesa, Municipio de San Bartolo Morelos	Estado de México	-99.73469444	19.7985
Mes1_3_S356001	MESA_I_3	MESA_I	La Mesa, Municipio de San Bartolo Morelos	Estado de México	-99.73469444	19.7985
Mes2_10_S366001	MESA_II_10	MESA_II	La Mesa, Municipio de San Bartolo Morelos	Estado de México	-99.73377778	19.79580556
Mes2_12_S351001	MESA_II_12	MESA_II	La Mesa, Municipio de San Bartolo Morelos	Estado de México	-99.73377778	19.79580556
Mes2_9_S343001	MESA_II_9	MESA_II	La Mesa, Municipio de San Bartolo Morelos	Estado de México	-99.73377778	19.79580556
Snag_1-1a_S305001	SAGU_1-1a	SAGU	Sin Agua, Municipio de Sahuayo	Michoacán	-102.826073	20.055996
Snag_1-1b_S294001	SAGU_1-1b	SAGU	Sin Agua, Municipio de Sahuayo	Michoacán	-102.826073	20.055996
Snag_1-1c_S329001	SAGU_1-1c	SAGU	Sin Agua, Municipio de Sahuayo	Michoacán	-102.826073	20.055996
Snjs_1-1a_S359001	SNJS_1-1a	SNJS	San Jose de Garcia, Municipio de Tepatitlán	Jalisco	-102.568534	20.683045
Snjs_1-1b_S295001	SNJS_1-1b	SNJS	San Jose de Garcia, Municipio de Tepatitlán	Jalisco	-102.568534	20.683045
Snjs_2-1a_S356001	SNJS_2-1a	SNJS	San Jose de Garcia, Municipio de Tepatitlán	Jalisco	-102.568534	20.683045
Snjs_2-1b_S298001	SNJS_2-1b	SNJS	San Jose de Garcia, Municipio de Tepatitlán	Jalisco	-102.568534	20.683045
Snjs_3-1a_S324001	SNJS_3-1a	SNJS	San Jose de Garcia, Municipio de Tepatitlán	Jalisco	-102.568534	20.683045

Snjs_3-1c_S364001	SNJS_3-1c	SNJS	San Jose de Garcia, Municipio de Tepatitlán	Jalisco	-102.568534	20.683045
Snjs_3-1d_S378001	SNJS_3-1d	SNJS	San Jose de Garcia, Municipio de Tepatitlán	Jalisco	-102.568534	20.683045
Snjs_3-1e_S314001	SNJS_3-1e	SNJS	San Jose de Garcia, Municipio de Tepatitlán	Jalisco	-102.568534	20.683045
Tequ_1-1_S451001	ARAN_1-1	ARAN	Arandas, Municipio de Arandas	Jalisco	-102.26736944	20.665925
Tequ_1-7a_S297001	ARAN_1-7a	ARAN	Arandas, Municipio de Arandas	Jalisco	-102.26736944	20.665925
Tequ_1-7b_S358001	ARAN_1-7b	ARAN	Arandas, Municipio de Arandas	Jalisco	-102.26736944	20.665925
Tequ_1-7c_S391001	ARAN_1-7c	ARAN	Arandas, Municipio de Arandas	Jalisco	-102.26736944	20.665925
Tequ_1-8a_S406001	ARAN_1-8a	ARAN	Arandas, Municipio de Arandas	Jalisco	-102.26736944	20.665925
Tequ_2-2a_S344001	ARAN_2-2a	ARAN	Arandas, Municipio de Arandas	Jalisco	-102.26736944	20.665925
Tequ_2-2b_S350001	ARAN_2-2b	ARAN	Arandas, Municipio de Arandas	Jalisco	-102.26736944	20.665925
Tequ_2-3b_S410001	ARAN_2-3b	ARAN	Arandas, Municipio de Arandas	Jalisco	-102.26736944	20.665925
Tequ_3-4_S473001	ARAN_3-4	ARAN	Arandas, Municipio de Arandas	Jalisco	-102.26736944	20.665925
Tequ_4-2a_S354001	ARAN_4-2a	ARAN	Arandas, Municipio de Arandas	Jalisco	-102.26736944	20.665925
Tequ_4-2b_S448001	ARAN_4-2b	ARAN	Arandas, Municipio de Arandas	Jalisco	-102.26736944	20.665925
Tequ_4-2c_S400001	ARAN_4-2c	ARAN	Arandas, Municipio de Arandas	Jalisco	-102.26736944	20.665925
Tequ_4-4a_S422001	ARAN_4-4a	ARAN	Arandas, Municipio de Arandas	Jalisco	-102.26736944	20.665925
Tequ_4-4b_S468001	ARAN_4-4b	ARAN	Arandas, Municipio de Arandas	Jalisco	-102.26736944	20.665925
Zita_1-1_S304001	ZITA_1-1	ZITA	Zitácuaro, Municipio de Heroica Zitácuaro	Michoacán	-100.396667	19.49166667

Table S2. Basic summary statistics for the localities analyzed in this study~~for the localities~~.

Locality	H_E	H_O	F_{IS}	N
BUEN	0.1937506	0.2058712	-0.0655	11
SNJS	0.1999692	0.2122008	-0.0616	8
ARAN	0.2365699	0.2419543	-0.0241	14

ANTE	0.2339699	0.2459685	-0.0539	7
SAGU	0.204843	0.2176689	-0.0664	3
COMA_II	0.2653203	0.2814152	-0.0606	12
COEN	0.2493989	0.2766499	-0.1107	7
ERON	-	0.2665478	-	1
COMA_I	0.2609418	0.279168	-0.071	14
CERR	0.2119145	0.2751895	-0.3005	7
ZITA	-	0.2108887	-	1
MESA_I	0.160597	0.3120832	-0.9331	2
MESA_II	0.1604727	0.3089685	-0.9255	3
ATZI	-	0.2817712	-	1

Table S3. ~~Paired~~Pairwise FST among localities.

	**COMA_I**	**COMA_II**	**ANTE**	**BUEN**	**COEN**	**CERR**	**MESA_I**	**MESA_II**	**SAGU**	**SNJS**
**COMA_II**	0.0609									
**ANTE**	0.1267	0.113								
**BUEN**	0.1709	0.1593	0.174							
**COEN**	0.0831	0.0782	0.1391	0.1888						
**CERR**	0.1565	0.1533	0.2067	0.2627	0.1723					
**MESA_I**	0.1688	0.1615	0.221	0.2768	0.1956	0.2787				
**MESA_II**	0.1967	0.1898	0.2478	0.299	0.2229	0.2962	**0.0004***			
**SAGU**	0.1754	0.1627	0.1658	0.2415	0.1965	0.2767	0.3075	0.3321		
**SNJS**	0.175	0.1627	0.2005	0.2233	0.1935	0.2652	0.2819	0.3037	0.2566	
**ARAN**	0.1196	0.1055	0.1357	0.1694	0.1359	0.2036	0.2062	0.2332	0.1917	0.1333

*****not significant distinct from zero

Table S4. ~~Paired~~Pairwise FST values among genetic groups, all comparisons were statistically significant different from zero.

	N~~O.~~W~~E~~	S~~O.~~W~~E~~	CENT	EAST
NO.WE	--			
SO.WE	0.0880	--		
CENT	0.0715	0.0791	--	
EAST	0.2033	0.2181	0.1626	--
